# MOBILE-BASED MULTIDOMAIN LIFESTYLE INTERVENTION AND HEALTH PROMOTION IN OLDER KOREAN AMERICANS

**DOI:** 10.1093/geroni/igac059.768

**Published:** 2022-12-20

**Authors:** Junhyoung Kim

**Affiliations:** Indiana University, Bloomington, Indiana, United States

## Abstract

Prior studies have suggested that the nature-based virtual reality (VR) program serves as a therapeutic tool to increase health and wellbeing among older adults. While much research on virtual reality indicates their health benefits among older adults, there are research gaps in the current literature on the nature-based VR experiences of older Korean Americans. The objective of this study was to gather preliminary information on the health outcomes of the program as well as challenges to participation. Based on in-depth interviews with five participants, three main salient themes as positive outcomes emerged: (a) enjoyment, (b) stress reduction, and (c) cognitive function. On the other hand, there were three main challenges that participants experienced: (a) challenges in the immersion experience itself, (b) difficulties with the controllers, and (c) cybersickness. This finding suggests that the nature-based virtual reality program can serve older immigrant population as a therapeutic tool to increase their mental and cognitive health.

